# The Role of Glutamatergic and Dopaminergic Neurons in the Periaqueductal Gray/Dorsal Raphe: Separating Analgesia and Anxiety

**DOI:** 10.1523/ENEURO.0018-18.2019

**Published:** 2019-02-19

**Authors:** Norman E. Taylor, JunZhu Pei, Jie Zhang, Ksenia Y. Vlasov, Trevor Davis, Emma Taylor, Feng-Ju Weng, Christa J. Van Dort, Ken Solt, Emery N. Brown

**Affiliations:** 1 University of Utah, Salt Lake City 84112, UT; 2 Massachusetts Institute of Technology, Cambridge 02139, MA; 3 Brigham Young University, Provo 84602, UT; 4 University of Massachusetts, Lowell 01854, MA; 5 Massachusetts General Hospital, Boston 02114, MA; 6 Picower Institute for Learning and Memory, MIT, Cambridge, MA 02139

**Keywords:** analgesia, anxiety, dopamine, DREADDs, periaqueductal gray

## Abstract

The periaqueductal gray (PAG) is a significant modulator of both analgesic and fear behaviors in both humans and rodents, but the underlying circuitry responsible for these two phenotypes is incompletely understood. Importantly, it is not known if there is a way to produce analgesia without anxiety by targeting the PAG, as modulation of glutamate or GABA neurons in this area initiates both antinociceptive and anxiogenic behavior. While dopamine (DA) neurons in the ventrolateral PAG (vlPAG)/dorsal raphe display a supraspinal antinociceptive effect, their influence on anxiety and fear are unknown. Using DAT-cre and Vglut2-cre male mice, we introduced designer receptors exclusively activated by designer drugs (DREADD) to DA and glutamate neurons within the vlPAG using viral-mediated delivery and found that levels of analgesia were significant and quantitatively similar when DA and glutamate neurons were selectively stimulated. Activation of glutamatergic neurons, however, reliably produced higher indices of anxiety, with increased freezing time and more time spent in the safety of a dark enclosure. In contrast, animals in which PAG/dorsal raphe DA neurons were stimulated failed to show fear behaviors. DA-mediated antinociception was inhibitable by haloperidol and was sufficient to prevent persistent inflammatory pain induced by carrageenan. In summary, only activation of DA neurons in the PAG/dorsal raphe produced profound analgesia without signs of anxiety, indicating that PAG/dorsal raphe DA neurons are an important target involved in analgesia that may lead to new treatments for pain.

## Significance Statement

Clinicians have long had the goal of separating analgesia from anxiety when using deep brain electrical stimulation of the periaqueductal gray (PAG) for difficult to treat pain. Here, we show that selective activation of dopamine (DA) neurons within the PAG produces analgesia without other behavioral effects, while stimulating glutamate neurons mediates stress-induced anxiety and analgesia. Our results suggest that DA agonists may represent a novel class of analgesic drugs and elucidate target neurons that could mediate their effect.

## Introduction

The midbrain periaqueductal gray (PAG) plays a significant role in pain and analgesia, fear and anxiety, and cardiovascular control (Behbehani, 1995; Millan, 2002; Tovote et al., 2016). Electrical stimulation of the PAG and periventricular gray in both animals (Reynolds, 1969) and humans (Hosobuchi et al., 1977; Richardson and Akil, 1977) produces profound antinociception, and has been used clinically to alleviate difficult to treat pain. While some patients receive considerable analgesic benefit (Bittar et al., 2005), the use of electrical stimulation of the PAG as a clinical analgesic modality has been limited because of fear and anxiety side effects in some patients (Shapira et al., 2006). A long-standing question in clinical pain research is whether there is a way to separate the generation of analgesia from anxiety when targeting the PAG (Fardin et al., 1984).

Early studies examined the effects of location within the PAG on antinociceptive and anxiety behaviors. Both electrical and pharmacologic stimulation of dorsal and lateral areas of the PAG produce flight reactions such as running and jumping, while there appear to be purely analgesic zones within the ventrolateral PAG (vlPAG) and dorsal raphe (Fardin et al., 1984). However, this is not a consistent finding, as freezing behavior can be elicited by pharmacologic stimulation of the vlPAG (Morgan et al., 1998; Kim et al., 2013), suggesting that a specific neuron type within the vlPAG might be responsible for the purely analgesic effect.

The vlPAG is comprised of a diverse population of neurons including glutamatergic, GABAergic, serotonergic and dopaminergic cells. GABAergic neurons in the vlPAG have particularly dense mu opioid receptor expression, and opioids are able to inhibit vlPAG GABAergic interneurons (Vaughan and Christie, 1997; Vaughan et al., 1997) leading to activation of glutamatergic projections to the rostral ventral medulla (RVM; Wiklund et al., 1988; Beitz, 1990; Reichling and Basbaum, 1990). In addition, a subset of GABAergic neurons also project directly to the RVM (Morgan et al., 2008). This classic descending pain inhibition circuit terminates at the dorsal horn of the spinal cord and powerfully modulates ascending nociceptive signals. However, in addition to their antinociceptive effects, opioids and GABA antagonists microinjected into the vlPAG also produce fear behaviors (Jacquet and Lajtha, 1974; Tomaz et al., 1988) and selective inhibition of local GABAergic vlPAG neurons induce freezing (Tovote et al., 2016). Therefore, vlPAG GABAergic neurons cannot be targeted to produce exclusive analgesia effects.

Glutamatergic neurons in the vlPAG are also involved in antinociception. Both optogenetic (Tovote et al., 2016) and chemogenetic (Samineni et al., 2017) activation of vlPAG glutamatergic neurons produce analgesia, and project to the RVM in the descending pain inhibition circuit. However, they also serve as the output neurons from the vlPAG in the circuit mediating fear and anxiety responses to a threat. GABAergic inputs to the vlPAG from the central nucleus of the amygdala (CEA) and the posterior hypothalamus (Falconi-Sobrinho et al., 2017) mediate glutamatergic outputs to pre-motor targets in the magnocellular nucleus of the medulla which mediate freezing (Miguel and Nunes-de-Souza, 2006; Tovote et al., 2016). Therefore, vlPAG glutamatergic neurons also cannot be targeted to produce exclusive analgesia effects.

One potential target could be vlPAG/dorsal raphe dopamine (DA) neurons. There is an understudied population of DA neurons in the vlPAG/dorsal raphe which is thought to exert analgesic effects (Suckow et al., 2013). Chemical lesion of PAG DA neurons attenuates opioid-induced antinociception (Flores et al., 2004) while local injection of the DA agonist apomorphine into the vlPAG produces antinociception (Meyer et al., 2009; Schoo et al., 2017). Furthermore, optogenetic activation of vlPAG/dorsal raphe DA neurons results in DA release in the bed nucleus of the stria terminalis (BNST) and a supraspinal antinociceptive effect (Li et al., 2016). The effects of vlPAG/dorsal raphe DA neurons on fear and anxiety behaviors, however, are completely unknown. In this study, we use a chemogenetic approach to specifically target dopaminergic and glutamatergic neurons in the vlPAG to test the hypothesis that dopaminergic neurons are antinociceptive without being anxiogenic.

## Materials and Methods

### Experimental animals

Adult male DAT-Cre (The Jackson Laboratory, stock number 006660) and vGlut2-ires-Cre mice (The Jackson Laboratory, stock number 016963), weighing 20–25 g, were used for all experiments. Mice were kept on a 12/12 h light/dark cycle (lights on at 7 A.M., lights off at 7 P.M.) with *ad libitum* access to food and water. Mice had a minimum of three weeks to recover after surgery, and at least 3 d of rest were provided after each experiment. All animal procedures were reviewed and approved by the authors Animal Care and Use Committee.

### Drugs

The following drugs were used in this study: the designer receptors exclusively activated by designer drugs (DREADD) activator clozapine-N-oxide (CNO; C0832, Sigma-Aldrich), SCH-23390 (D1 receptor antagonist, D054, Sigma-Aldrich), raclopride (D2 receptor antagonist, R121, Sigma-Aldrich), haloperidol (non-specific DA receptor antagonist, 67457-426-12, Mylan), and the inflammatory agent carrageenan (C1013, Sigma-Aldrich). All drugs were diluted in saline.

### Chemogenetic manipulation

To induce the expression of DREADDs in the vlPAG, 300nl of adeno-associated virus (AAV) carrying either the AAV8-hSyn-DIO-hM3D(Gq)-mCherry (excitatory, hM3), AAV8-hSyn-DIO-hM4D(Gi)-mCherry (inhibitory, hM4) vectors, or a virus containing only a fluorescent tag without a receptor (AAV8-hSyn-DIO-mCherry, UNC Vector Core), were injected bilaterally at –4.7 mm anterior/posterior, ±0.5 mm lateral, and –2.75 mm dorsal/ventral to bregma. Briefly, mice were anesthetized with 2% isoflurane and placed in a stereotaxic frame (David Kopf Instruments). An incision was made in the skin, and craniotomies were made above the target region. The injections were performed using a motorized stereotaxic injector (Stoelting) and the mice recovered for three weeks to allow optimal viral expression.

### Experimental procedures

#### Nociceptive behavior testing

To evaluate nociception, thermal withdrawal latencies and mechanical withdrawal thresholds were assayed as previously described (Samineni et al., 2017). The Hargreaves test was performed to evaluate heat sensitivity thresholds, measuring latency of withdrawal to a radiant heat source (IITC Life Science, Model 390). The radiant heat was applied to the plantar surface of both hind paw and the latency to evoke a withdrawal was measured. Three to five replicates, measured every 5 min over 20 min, were acquired per hind paw per mouse, and the values for both paws were averaged. At least 2 d later, von Frey filaments (Stoetling Co.) were used to evaluate the mechanical nociceptive threshold. Filaments were applied, also to the plantar surface of both hind paws of the mice, five times, increasing thickness until a withdrawal response was observed three times. The force of the corresponding filament was recorded as the mechanical nociceptive threshold for each mouse.

For the nociceptive behavior evaluations, mice were habituated to the assessment chambers starting two weeks after viral injections. The baseline nociceptive thresholds were determined on the 3rd week postsurgery. Following the baseline measurements, mice received intraperitoneal injection of saline, CNO alone (1 mg/kg), CNO plus SCH-23390 (0.5 mg/kg), CNO plus raclopride (0.5 mg/kg), or CNO plus haloperidol (0.3 mg/kg), in a blinded fashion. Then, the mice were placed back within their individual Plexiglas compartments for 60 min before beginning behavioral assessment. Paw withdrawal latencies or thresholds were collected between the first and second hour after injection. The minimal dose of CNO needed to activate the DREADDs, and the minimal dose of antagonists required to achieve peak effect, were determined by previous experiments (data not shown).

#### Fear behavior testing

After thermal nociception testing, mice were individually placed into the center of an open field test environment of dark acrylic plastic (40 × 40 × 40 cm), under dim lit conditions with the same experimenter in the room. Their movements were recorded for 5 min using a USB camera and video tracking system (Any-Maze, Stoetling Co.). At least 2 d later, following mechanical nociception testing, mice were individually placed into the dark side of a light-and-dark test environment of acrylic plastic (40 × 40 × 40 cm), and their movements within the light areas of the box were recorded for 5 min using the video tracking system.

#### Inflammatory pain model

To evaluate the role of dopaminergic vlPAG/dorsal raphe neurons in pain produced by inflammation, 25 µl of a 20 mg/ml solution of carrageenan, dissolved in saline, was injected subcutaneously into the plantar surface of the hind paw of DAT-cre and vGlut2-cre mice, as previously described (Hargreaves et al., 1988). Three hours later, mice received intraperitoneal injection of CNO, and 1 h later, paw withdrawal latency to a thermal stimulus (Hargreaves test) was evaluated.

### *In vitro* electrophysiology

#### Patch clamp slice preparation

Identified mice were anesthetized with isoflurane and swiftly decapitated. The brains were harvested and rapidly immersed in ice cold carbonated (95% O_2_ and 5% CO_2_)-cutting solution composed of 105 mM *N-*methyl-D-glucamine, 26 mM NaHCO_3_,15 mM glucose, 10 mM MgCl_2_, 2.5 mM KCl, 1.24 mM NaH_2_PO_4_, 0.5 mM CaCl_2_, and 1 mM Na ascorbate at an osmolality of 300 mOsm. The pH of this solution was titrated to 7.3 with HCl. The brain tissue was subsequently blocked and sliced into 350-μm coronal sections with a vibrating blade microtome (VT1200, Leica). Slices that contained the vlPAG were relocated into an incubation chamber filled with warm (32°C) carbonated cutting solution for 10 min followed by recovery solution (32°C) containing 50% cutting solution and 50% artificial CSF (ACSF) for 20 min. The coronal brain slices were then held in a holding chamber containing carbonated ACSF (pH 7.3) composed of 119 mM NaCl, 26 mM NaHCO_3_, 2.5 mM KCl, 2.5 mM CaCl_2_, 1.3 MgCl_2_, and 10 mM glucose at an osmolarity of 300 mOsm at room temperature (∼23°C) for at least 1 h before resuming patch experiments.

### Electrophysiology recordings

Following at least 1 h of recovery, the coronal brain slices were transferred to a recording chamber circulated with carbonated ACSF in room temperature (∼23°C) at a flow rate of 2 ml/min. A borosilicate glass pipette (tip resistance between 3 and 6 MΩ) was filled with a solution composed of 130 mM K-gluconate, 10 mM KCl, 10 mM HEPES, 4 mM Mg-ATP, 0.2 mM EGTA, and 0.5 mM Na-GTP at an osmolarity of 290 mOsm (pH 7.25). CNO-evoked currents were recorded in voltage-clamp mode with membrane potential held at −70 mV, and CNO-evoked spikes were recorded in current-clamp mode with a holding potential at around −55 mV. DREADD-mCherry-expressing cells were identified by mCherry expression, and cells were stimulated using 10 µM CNO. Data, acquired with an Axon Multiclamp 700B amplifier and a Digidata 1440 digitizer (Molecular Devices), were analyzed using Axon Clampfit. Recordings with access resistance >25 MΩ or with changes in access resistance >15% were discarded.

### Immunohistochemistry

After all experiments were completed, viral expression and localization was verified via histologic analysis. Dat-cre animals were perfused with phosphate buffered saline followed by neutral buffered formalin. The brains were postfixed in formalin overnight, and sliced at 60 μm using a Leica VT1200 S vibratome (Leica Microsystems Inc.). Specific expression of DREADDs in DA neurons was confirmed by colocalization of mCherry (from AAV expression) with immunohistochemical staining for tyrosine hydroxylase (TH), a marker of DA neurons (mouse anti-TH, 1:1000 dilution, Millipore catalog #MAB318), using the secondary antibody of goat anti-mouse conjugated to Alexa Fluor 488 (1:200 dilution, catalog #A-11001, Invitrogen). Cells were counterstained with 4’,6-diamidino-2-phenylindole (DAPI; catalog #H-1200, Vectashield) for nuclear visualization. Images were taken with a Zeiss Axio M2 microscope (Zeiss). Confirmation of viral expression in the correct brain region was performed by comparing images to a Mouse Brain Atlas (Paxinos et al., 2001).

### Fluorescent *in situ* hybridization (FISH)

Brains were swiftly harvested and immediately flash frozen in a beaker filled with a bilayer of 1-methylbutane and 1-bromobutane on dried iced and subsequently stored in –80°C. The brains were sectioned on a cryostat and mounted on Superfrost Plus Gold slides (25 × 75 mm, Erie Scientific). One hour before sectioning, brains and slides were equilibrated to –20°C in the cryostat. The specified brains were serially sectioned coronally at 12 µm and mounted onto slides via the warmth of the hand. The mounted specimens were dried for 1 h inside the cryostat then stored at –80°C. Double-label FISH was performed using RNAScope Manual Fluorescent Multiplex kit User Manual specified for Fresh Frozen Tissue (Advanced Cell Diagnostics). Slides were fixed in 4% paraformaldehyde (PFA) at 4°C, serially dehydrated, washed twice in phosphate buffered saline pH 7.38, and pretreated with protease IV solution for 30 min. Specimens were then incubated with target probes for mouse vglut2 (*slc17a6* target region 1986–2998, catalog #319171, Advanced Cell Diagnostics) and mcherry (*mcherry* target region 23–681, catalog #431201-C2, Advanced Cell Diagnostics). Next, the slides underwent four serial amplification incubations the last of which contained fluorescent probes (Alexa Fluor 488 and Atto 550, catalog #320850, Advanced Cell Diagnostics Part of Florescent Multiplex kit) individually targeted to the slc17a6 and mcherry probes. Finally, the slides were mounted with ProLong Diamond Antifade Mountant with DAPI (catalog #P36962, Invitrogen). Images were taken with a Zeiss Imager M2 microscope (Zeiss).

### Statistical analysis

**Figure 1.**
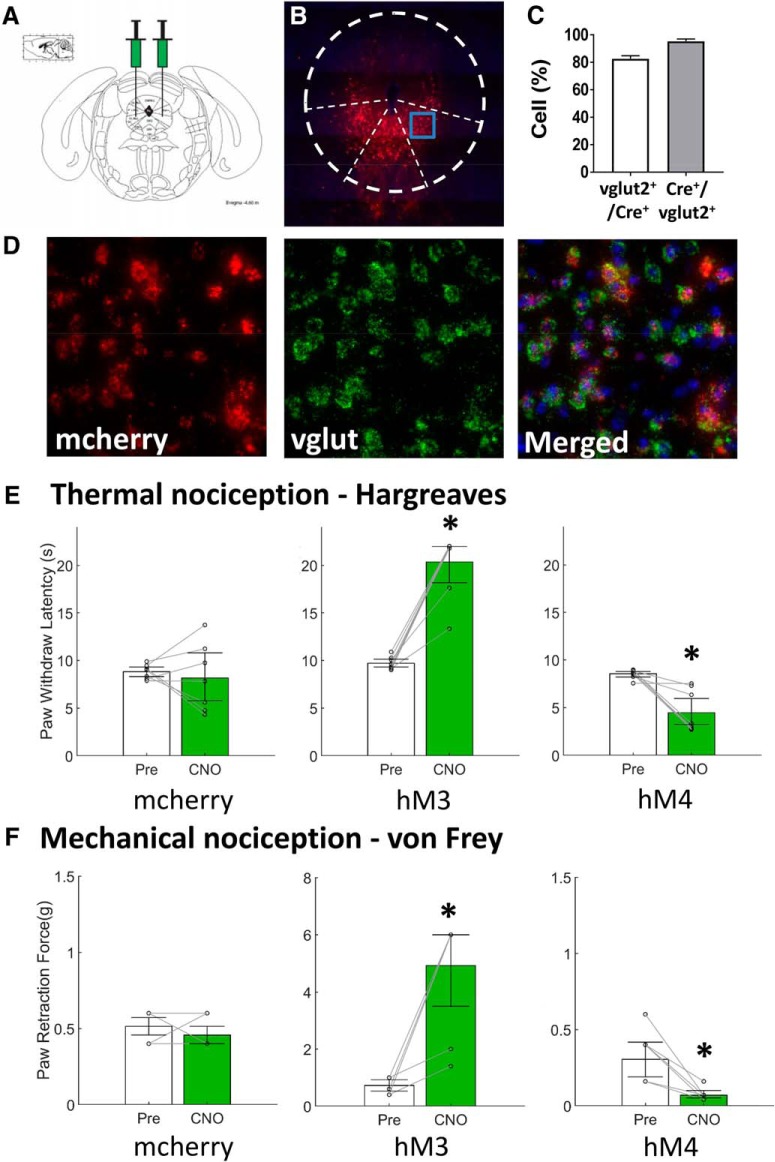
Glutamatergic neurons in the vlPAG produce antinociception ***A***, Glutamatergic neurons were targeted by local vlPAG injection of AAV in transgenic mice that expressed Cre under control of the vesicular glutamate transporter 2 gene (vGlut2-cre). ***B***, DREADDs expression in the vlPAG/dorsal raphe of vlgut2-cre mice (RNA-FISH). ***C***, 83 ± 2% of green-labeled vGlut2^+^ transcripts in the vlPAG and dorsal raphe colocalized with mcherry-labeled neurons expressing vGlut2 RNA, and 95 ± 2% of mcherry-labeled vGlut2 cre-expressing neurons colocalized with green-labeled vGlut2^+^ transcripts. ***D***, DREADDs are expressed in vlPAG/dorsal raphe vGlut2^+^ neurons as demonstrated by colocalized expression in the merged image. ***E***, ***F***, Bar graphs represent the median value of the data, while the error bars are the 95% CI. White bars indicate nociceptive testing before intraperitoneal CNO injections, while green bars indicate nociceptive testing 1 h after CNO administration. Pair-wise comparisons with a Wilcoxon signed-rank test indicated no significant behavioral difference in mCherry animals (*n* = 7) after CNO treatment. CNO activation of vlPAG glutamate neurons (hM3, *n* = 8) produced analgesia as indicated by increased paw withdrawal latencies to thermal (*p* = 0.0078) and mechanical stimuli (*p* = 0.0002) in vGlut2-cre mice, while inhibition (hM4, *n* = 8) caused increased sensitivity to both thermal (*p* = 0.0078) and mechanical stimuli (*p* = 0.0008). *p < 0.05.

**Figure 2.**
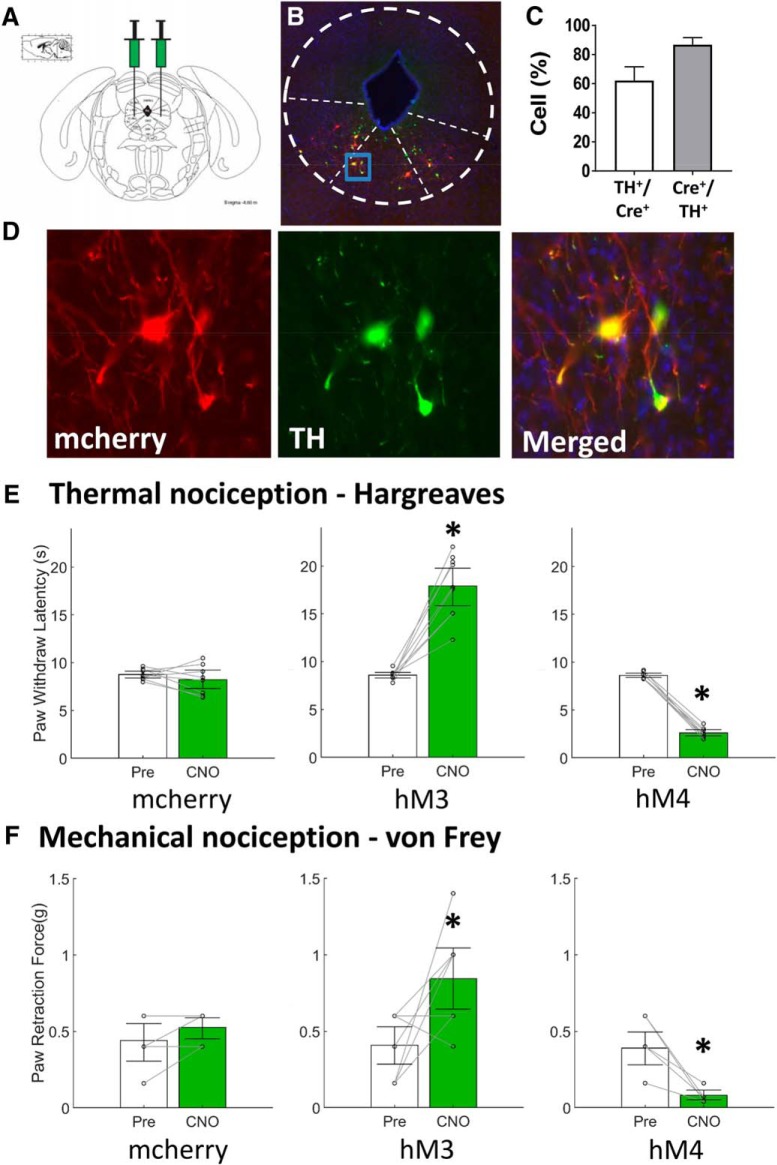
Dopaminergic neurons in the vlPAG produce antinociception. ***A***, Dopaminergic neurons were targeted by local vlPAG injection DREADD containing AAV into transgenic mice expressing Cre under control of the DA transporter gene (DAT-cre). ***B***, DREADDs expression in the vlPAG/dorsal raphe of DAT-cre mice (immunohistochemistry). ***C***, 61 ± 10% of green-labeled TH neurons in the vlPAG and dorsal raphe colocalized with mcherry-labeled neurons expressing DAT protein, and 84 ± 8% of mcherry-labeled DAT cre-expressing neurons colocalized with green-labeled TH containing neurons. ***D***, DREADDs are expressed in vlPAG/dorsal raphe TH^+^ neurons as demonstrated by colocalized expression in the merged image. ***E***, ***F***, Bar graphs represent the median value of the data, while the error bars are the 95% CI. White bars indicate nociceptive testing before intraperitoneal CNO injections while green bars indicate nociceptive testing 1 h after CNO administration. Pair-wise comparisons with a Wilcoxon signed-rank test indicated no significant behavioral difference in mcherry animals (*n* = 8) after CNO treatment. CNO activation of vlPAG DA neurons (hM3, *n* = 9) produced analgesia, with increased paw withdrawal latencies to both thermal (paired *t* test, df8, *p* < 0.0001) and mechanical stimuli (Wilcoxon signed rank, df8, *p* = 0.0313) in DAT-cre mice, while inhibition (hM4, *n* = 8) caused a significant decrease in paw withdrawal latencies to both thermal (paired *t* test, df7, *p* < 0.0001) and mechanical stimuli (Wilcoxon signed rank, df7, *p* = 0.0078). *p < 0.05.

GraphPad Prism 7.02 was used to perform statistical analysis. The Shapiro–Wilk test was first performed on each set of data to test for normality. To guard against type 1 error, a one-way ANOVA was applied to data determined to be normally distributed while a Kruskal–Wallis test was applied to the remaining groups of non-parametric data. As comparisons were made between pre-treated and post-treated conditions in the same mouse in the Hargreaves and von Frey data presented in Figures 1, 2, pair-wise comparisons were made using either a paired *t* test for the normally distributed DAT-cre mice Hargreaves data, or the Wilcoxon signed rank test for the remaining paired nonparametric data. Comparisons of the open field and light/dark test endpoints were made in different groups of animals; therefore, a one-way ANOVA was performed on each group of animals of the same strain whose data were parametric followed by a Dunnett’s multiple comparison test, while a Kruskal–Wallis test was followed by a Dunn’s multiple comparisons test in non-parametric data; *p* < 0.05 was considered significant in all cases. In addition, differences between experimental and control conditions were calculated, and bootstrapped 95% confidence intervals (CIs) were generated using MATLAB as confirmation of statistical significance. If the CI’s did not include 0, the difference between the experimental and control conditions was deemed to be statistically significant. Data are reported in the text as the median difference between experimental and control conditions with 95% CI. Figures include the raw data as points, with bar graphs depicting the median value with 95% CIs.

## Results

To determine the neurons within the PAG that could selectively produce antinociceptive behavioral phenotypes, we used a chemogenetic approach to specifically manipulate the activity of glutamatergic and dopaminergic neurons in the vlPAG and dorsal raphe. Glutamatergic neurons were targeted by local injection of AAV into the vlPAG of transgenic mice that expressed Cre under control of the vesicular glutamate transporter 2 gene (vGlut2-cre; Fig. 1*A*). Anesthetized vGlut2-cre mice received targeted injections of AAV carrying an excitatory DREADD (AAV8-hM3Dq, *n* = 8 mice/group), an inhibitory DREADDs construct (AAV8-hM4Di, *n* = 8 mice/group) or a virus only containing the mCherry fluorescent tag (*n* = 8). FISH demonstrated robust DREADD expression restricted to glutamate neurons in the the vlPAG and dorsal raphe (Fig. 1*B*,*D*). We observed 83 ± 2% of green-labeled vGlut2^+^ transcripts in the vlPAG and dorsal raphe colocalized with mcherry-labeled neurons expressing vGlut2 RNA, and 95 ± 2% of mcherry-labeled vGlut2 cre-expressing neurons colocalized with green-labeled vGlut2^+^ transcripts as shown in Figure 1*C*. Based on this histologic examination, no animals were excluded in the behavioral analysis. The transfection specificity and sensitivity were similar to values previously reported.

As shown in Fig. 1*E*, there was no significant difference between paw withdrawal latency to a thermal stimulus measured before and after intraperitoneal administration of the normally inert ligand CNO (–0.09 s, 95% CI [–1.56, 1.50 s], Wilcoxon signed rank, df7, *p* = 0.6875). Likewise, there was no change in paw withdrawal threshold to a mechanical nociceptive stimulus between control animals before and after intraperitoneal CNO (0 g, 95% CI [–0.14, 0.0 g], Wilcoxon signed rank, df7, *p* = 0.592), as shown in Figure 1*F*. CNO activation of vlPAG glutamate neurons increased both paw withdrawal latency (12.06 s, 95% CI [7.56, 12.66 s], Wilcoxon signed rank, df7, *p* = 0.0078) and mechanical nociceptive threshold (5 g, 95% CI [2.66, 5.23 g], Wilcoxon signed rank, df7, *p* = 0.0002) in vGlut-2-cre mice, while inhibition decreased paw withdrawal latencies (–6.85 s, 95% CI [–7.83, –3.12 s], Wilcoxon signed rank, df6, *p* = 0.0078) and thresholds (–0.18 g, 95% CI [–0.35, –0.14 g], Wilcoxon signed rank, df6, *p* = 0.0008).

Recently, it was discovered that CNO does not enter the brain after systemic injection but, rather, is rapidly converted to clozapine which binds to DREADDs with high affinity and potency and is responsible for the *in vivo* effect (Gomez et al., 2017). It is therefore significant that administration of CNO to mice expressing the mcherry tag but lacking DREADDs produced no significant difference in paw withdrawal latencies or paw withdrawal thresholds, demonstrating the absence of a non-specific CNO/clozapine effect.

Mice expressing Cre recombinase under the transcriptional control of the DA transporter promoter (DAT-cre mice) were similarly prepared, with virus containing either excitatory (hM3), inhibitory (hM4), or the mCherry tag only targeted to DA neurons in the vlPAG and dorsal raphe (Fig. 2*A*). Immunohistochemistry demonstrated robust DREADD expression restricted to the vlPAG and dorsal raphe that colocalized with neurons expressing tyrosine hydoxylase (Fig. 2*B*,*D*). We observed 61 ± 10% of green-labeled TH neurons in the vlPAG and dorsal raphe colocalized with mcherry-labeled neurons expressing DAT protein, and 84 ± 8% of mcherry-labeled DAT cre-expressing neurons colocalized with green-labeled TH containing neurons (Fig. 2*C*).

As shown in Figure 2*E*, there was no change in thermal sensitivity (paw withdrawal latency; –0.85 s, 95% CI [–1.84, 0.68 s], paired *t* test, df7, *p* = 0.0.363) nor in mechanical allodynia (Fig. 2*F*; paw withdrawal thresholds; 0 g, 95% CI [0, 0.22 g], Wilcoxon signed rank, df7, *p* = 0.25) in mCherry control mice before and after injection with CNO. CNO activation of vlPAG DA neurons (hM3, *n* = 9) reduced both the thermal (9.81 s, 95% CI [6.39, 12.10 s], paired *t* test, df8, *p* < 0.0001) and mechanical sensitivity (0.44 g, 95% CI [0.16, 0.72 g], Wilcoxon signed rank, df8, *p* = 0.0313) in DAT-cre mice, while inhibition (hM4, *n* = 8) caused a significant increase in both thermal (–5.96 s, 95% CI [–6.37, –5.48 s], paired *t* test, df7, *p* < 0.0001) and mechanical sensitivity (–0.29 g, 95% CI [–0.53, –0.18 g], Wilcoxon signed rank, df7, *p* = 0.0078).

**Figure 3.**
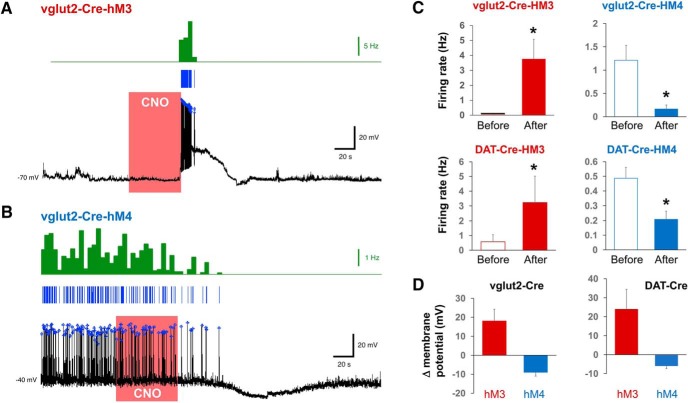
Functional characterization of hM3 and hM4 DREADDs in vlPAG/dorsal raphe neurons of vGglut2-Cre and DAT-cre mice. ***A***, Whole-cell current-clamp recording from an hM3Dq-expressing vlPAG neuron. Brief bath application of 10 µM CNO (red box) caused a transient depolarization and robust action potential firing in both vGlut2 and DAT neurons. Blue lines represent individual spike events. These were then aggregated into 5-s bins and the frequency plotted as shown in the green histogram. ***B***, Voltage trace showing that bath perfusion with 10 µM CNO caused prolonged membrane hyperpolarization and silencing of both vGlut and DAT vlPAG/dorsal raphe neurons. ***C***, Quantification of the CNO effects on neuron firing rate in grouped vGlut2 and DAT neurons (*n* = 4). ***D***, Quantification of the CNO effects on membrane potential (all values are mean ± SEM; *p < 0.05).

A separate group of animals was prepared for functional characterization of the hM3 and hM4 DREADDs. Three weeks after DREADD injection, acute coronal slices of the vlPAG/dorsal raphe (*n* = 4) were obtained from each group and were prepared for whole cell recordings to demonstrate fidelity of the expressed receptors. hM3-expressing vlPAG/dorsal raphe neurons were held at hyperpolarized membrane potentials and then were exposed to a bath application of 10 µM CNO (Fig. 3*A*). This caused a transient depolarization and robust action potential firing in both vGlut2 (3.7 Hz, 95% CI [1.2, 6.4 Hz]) and DAT expressing neurons (2.6 Hz, 95% CI [1.0, 7.0 Hz]). Neuronal inhibition by hM4Di was measured by first holding the cells with a depolarizing current, which elicited persistent action potential firing in both DAT and vGlut2 neurons, and then perfusing the bath with 10 µM CNO (Fig. 3*B*). This resulted in prolonged membrane hyperpolarization and decreased firing of both vGlut2 (0.2 Hz, 95% CI [0, 0.3 Hz]) and DAT expressing neurons (0.2 Hz, 95% CI [0.1, 0.3 Hz]; Fig. 3*C*). CNO significantly depolarized hM3Dq-expressing vGlut2 (Δ membrane potential 17.3 mV, 95% CI [6.8, 30.0 mV], *p* < 0.05) and DAT neurons (Δ membrane potential 20.6 mV, 95% CI [5.9, 45.2 mV], *p* < 0.05), while significantly hyperpolarizing hM4Di-expressing vGlut2 (Δ membrane potential –8.0 mV, 95% CI [–12.8, –6.3 mV], *p* < 0.05) and DAT containing neurons (Δ membrane potential –5.3 mV, 95% CI [–8.5, –3.9 mV], *p* < 0.05; Fig. 3*D*).

Interestingly, while levels of antinociception were significant and quantitatively similar when DA and glutamate neurons in the vlPAG/dorsal raphe were selectively stimulated, it was clear that the animals’ behavior was noticeably different. Activation of glutamatergic neurons in the vlPAG reliably triggered strong freezing behavior after administration of CNO, whereas activation of dopaminergic neurons did not. to quantify these differences in behavior, mice were exposed to a novel context under low-fear conditions, and had the level of anxiety in an open field test, and preferential exploration of a light area rather than a dark area, evaluated after selective stimulation of glutamatergic and dopaminergic vlPAG/dorsal raphe neurons.

**Figure 4.**
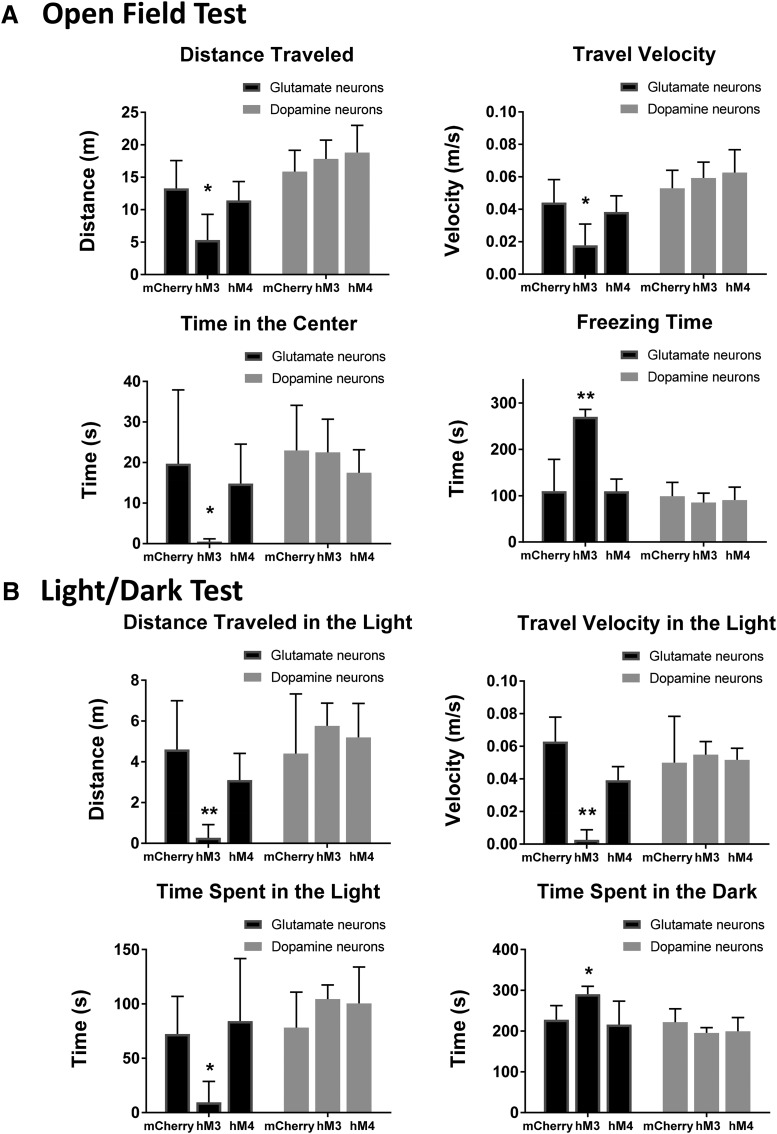
Glutamatergic neurons in the vlPAG drive fear responses, as assessed using open field and light/dark behavioral tests (mCherry = controls, hM3 = excitatory DREADD, hM4 = inhibitory DREADD). ***A***, Open field test. DREADD activation (hM3, *n* = 8) of vlPAG glutamate neurons in vglut-2-cre mice produced decrease in distance traveled, velocity of travel, and time spent in the center of an open field along with increase in the time spent frozen when compared with control mice, while inhibition (hM4, *n* = 8) had no effect on these endpoints (ANOVA with Dunnett’s multiple comparison test, *p < 0.05, **p < 0.001). In contrast, DREADD activation (hM3, *n* = 8) or inhibition (hM4, *n* = 8) of dopaminergic vlPAG/dorsal raphe neurons in DAT-cre mice had no effect on distance traveled, travel velocity, center time, or freezing time (one-way ANOVA). ***B***, In the light/dark test, DREADD activation (hM3, *n* = 8) of vlPAG/dorsal raphe glutamatergic neurons lead to decreased distance traveled, travel velocity, and time spent in the light side of the chamber as well as increased time spent in the dark, enclosed side of the chamber (Kruskal–Wallis with Dunn’s multiple comparison test, *p < 0.05, **p < 0.001). CNO inhibition of glutamatergic vlPAG/dorsal raphe neurons (hM4, *n* = 8) had no effect on these end points. In contrast, DREADD activation or inhibition of dopaminergic vlPAG/dorsal raphe neurons (hM3, *n* = 8) also had no effect on distance traveled, travel velocity, or time spent on the light side of the enclosure nor on time spent in the dark, enclosed side (Kruskal–Wallis).

As shown in Figure 4*A*, CNO activation of glutamatergic vlPAG/dorsal raphe neurons (hM3, *n* = 8) produced behaviors consistent with anxiety including decreased distance traveled (from 13.3 m, 95% CI [9.0–17.6 m] in the control, to 5.3 m, 95% CI [1.4–9.3 m] in hM3, ANOVA with Dunnett’s, df7, *p* = 0.0036), travel velocity (from 0.044 m/s, 95% CI [0.03–0.058 m/s] in the control, to 0.018 m/s, 95% CI [0.005–0.031 m/s] in hM3, ANOVA with Dunnett’s, df7, *p* = 0.0037), and time spent in the center of the open field (from 19.7 s, 95% CI [1.5–37.9 s] in the control, to 0.5 s, 95% CI [–0.1–1.2 s] in hM3, Kruskal–Wallis with Dunn’s, df7, *p* = 0.014) as well as increased freezing time (from 109.8 s, 95% CI [41.3–178.3 s] in the control, to 270.3 s, 95% CI [254.3–286.2 s] in hM3, ANOVA with Dunnett’s, df7, *p* = 0.0001). CNO inhibition of glutamatergic vlPAG/dorsal raphe neurons (hM4, *n* = 8) had no effect on these end points (11.4 m, 95% CI [8.5–14.4 m], ANOVA with Dunnett’s, df7, *p* = 0.624; 0.0.38 m/s, 95% CI [0.028–0.048 m/s], ANOVA with Dunnett’s, df7, *p* = 0.658; 14.8 s, 95% CI [5.1–24.5 s], Kruskal–Wallis with Dunn’s, df7, *p* > 0.999; 109.4 s, 95% CI [82.9–135.9 s], ANOVA with Dunnett’s, df7, *p* = 0.9997). DREADD activation or inhibition of dopaminergic vlPAG/dorsal raphe neurons (hM3, *n* = 8) also had no effect on distance traveled, travel velocity, center time or freezing time (distance traveled: 15.9 m, 95% CI [12.6–19.2 m] control, 17.8 m, 95% CI [14.9–20.7 m] hM3, 18.8 m, 95% CI [14.6–23.0 m] hM4, ANOVA, df7, *p* = 0.39; travel velocity: 0.053 m/s, 95% CI [0.042–0.064 m/s] control, 0.059 m/s, 95% CI [0.050–0.069 m/s] in hM3, 0.063 m/s, 95% CI [0.049–0.077 m/s] in hM4, ANOVA, df7, *p* = 0.40; time in the center: 23.0 s, 95% CI [11.9–34.1 s] control, 22.5 s, 95% CI [14.4–30.7 s] hM3, 17.5 s, 95% CI [11.8–23.1 s] hM4, ANOVA, df7, *p* = 0.52; freezing time: 98.9 s, 95% CI [68.9–128.9 s] control, 85.2 s, 95% CI [65.03–105.4 s] hM3, 90.5 s, 95% CI [62.6–118.4 s] hM4, ANOVA, df7, *p* = 0.68).

In the light/dark test, activation of vlPAG/dorsal raphe glutamatergic neurons lead to decreased distance traveled (from 4.6 m, 95% CI [2.2–7.0 m] in the control, to 0.3 m, 95% CI [–0.4–0.9 m] in hM3, Kruskal–Wallis with Dunn’s, df7, *p* = 0.0009), travel velocity (from 0.062 m/s, 95% CI [0.048–0.077 m/s] in the control, to 0.003 m/s, 95% CI [–0.004–0.009 m/s] in hM3, Kruskal–Wallis with Dunn’s, df7, *p* < 0.0001) and time spent in the light side of the chamber (from 72.3 s, 95% CI [37.7–106.9 s] in the control, to 9.5 s, 95% CI [–9.7–28.7 s] in hM3, Kruskal–Wallis with Dunn’s, df7, *p* = 0.011) as well as increased time spent in the dark, enclosed side of the chamber (from 227.7 s, 95% CI [193.1–262.3 s] in the control, to 290.5 s, 95% CI [271.3–309.7 s] in hM3, Kruskal–Wallis with Dunn’s, df7, *p* = 0.011) as show in Figure 4*B*. CNO inhibition of glutamatergic vlPAG/dorsal raphe neurons (hM4, *n* = 8) had no effect on these end points (3.1 m, 95% CI [1.8–4.4 m], Kruskal–Wallis with Dunn’s, df7, *p* = 0.890; 0.0.39 m/s, 95% CI [0.031–0.048 m/s], Kruskal–Wallis with Dunn’s, df7, *p* = 0.0974; 84.2 s, 95% CI [26.6–141.7 s], Kruskal–Wallis with Dunn’s, df7, *p* > 0.999; 215.9 s, 95% CI [158.3–273.4 s], Kruskal–Wallis with Dunn’s, df7, *p* > 0.999). DREADD activation or inhibition of dopaminergic vlPAG/dorsal raphe neurons (hM3, *n* = 8) also had no effect on distance traveled, travel velocity, or time spent on the light side of the enclosure nor on time spent in the dark, enclosed side (distance traveled: 4.4 m, 95% CI [1.5–7.3 m] control, 5.8 m, 95% CI [4.7–6.9 m] hM3, 5.2 m, 95% CI [3.5–6.9 m] hM4, Kruskal–Wallis, df7, *p* = 0.475; travel velocity: 0.05 m/s, 95% CI [0.022–0.078 m/s] control, 0.055 m/s, 95% CI [0.047–0.063 m/s] in hM3, 0.052 m/s, 95% CI [0.044–0.059 m/s] in hM4, ANOVA, df7, *p* = 0.885; time in the light: 78.2 s, 95% CI [45.5–110.8 s] control, 104.4 s, 95% CI [91.4–117.4 s] hM3, 100.5 s, 95% CI [66.9 to 134 s] hM4, Kruskal–Wallis, df7, *p* = 0.271; time in the dark: 221.8 s, 95% CI [189.2–254.5 s] control, 195.6 s, 95% CI [182.6–208.7 s] hM3, 199.6 s, 95% CI [166–233.1 s] hM4, Kruskal–Wallis, df7, *p* = 0.271).

In summary, vGlut2-cre animals consistently showed higher indices of anxiety, with increased freezing time and more time spent in the safety of the dark area when administered CNO, suggesting that vlPAG glutamatergic neuron activation mediated the anxiogenic effect. In contrast, DAT-cre mice with hM3 DREADD expression in the vlPAG/dorsal raphe failed to show anxiety behavior when administered CNO, suggesting that activation of DA neurons in this region is analgesic without being anxiogenic.

**Figure 5.**
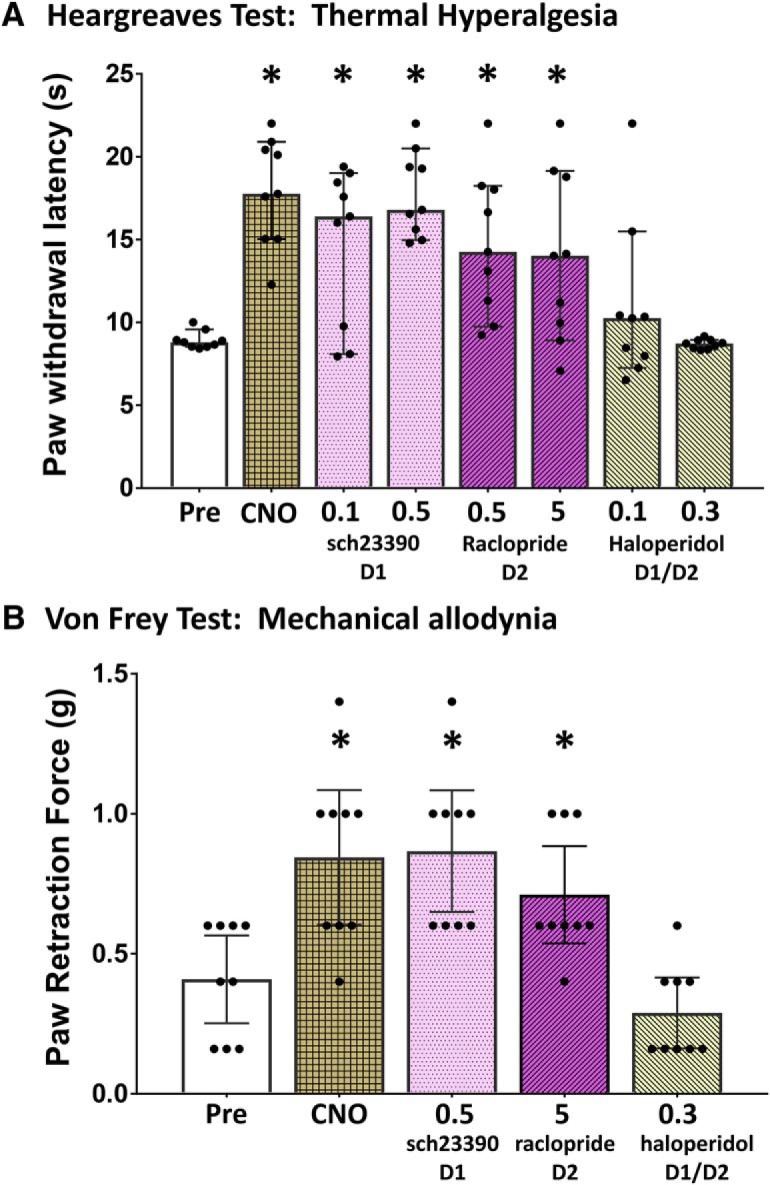
Haloperidol inhibits vlPAG dopaminergic neuron mediated analgesia. ***A***, ***B***, Changes in paw withdrawal latencies to a thermal test were significant (one-way ANOVA, *p* < 0.0001) as were changes in paw retraction force (Kruskal–Wallis, *p* < 0.0001). Pair-wise comparisons indicated that only the nonspecific DA receptor antagonist haloperidol (0.3 mg/kg) prevented the analgesia induced by activation of vlPAG DA neurons by CNO (1 mg/kg), as paw withdrawal latencies (paired *t* test, *p* = 0.413) and retraction forces (Wilcoxon signed rank, *p* = 0.371) showed no significant change from baseline. In contrast, treatment with the selective D1 receptor antagonist SCH-23390 (0.5 mg/kg) or the selective D2 receptor antagonist raclopride (0.5 mg/kg) were ineffective in preventing the analgesia. *p < 0.05.

To determine the receptors mediating the vlPAG/dorsal raphe dopaminergic antinociceptive effect, subtype selective DA receptor antagonists were administered systemically. Pretreatment with the selective D1 receptor antagonist SCH-23390 (0.5 mg/kg; 4.94 s, 95% CI [3.85, 9.57 s], paired *t* test, df8, *p* < 0.0001) or the selective D2 receptor antagonist raclopride (5 mg/kg; 1.15 s, 95% CI [3.35, 8.53 s], paired *t* test, df8, *p* = 0.0176) failed to prevent the increase in paw withdrawal latency exhibited by CNO activation of vlPAG DA neurons (9.81 s, 95% CI [6.39, 12.10 s], paired *t* test, df8, *p* < 0.0001). In contrast, the nonspecific DA receptor antagonist haloperidol (0.3 mg/kg; –0.20 s, 95% CI [–0.44, 0.21 s]) was effective in blocking the antinociceptive effect, resulting in no significant difference in paw withdrawal latency from baseline (paired *t* test, df8, *p* = 0.413; Fig. 5*A*). The results were similar when mice were exposed to a mechanical nociceptive stimulus (Fig. 5*B*), with only haloperidol effectively preventing vlPAG DA neuron-mediated antinociception (–0.24 g, 95% CI [–0.32, 0.08 g], Wilcoxon signed rank, *p* = 0.371). However, treatment with SCH-23390 (0.5 mg/kg; 0.44 g, 95% CI [0.29, 0.62 g], Wilcoxon signed rank, df7, *p* = 0.0078) or raclopride (0.5 mg/kg; 0.24 g, 95% CI [0.13, 0.50 g], Wilcoxon signed rank, df7, *p* = 0.0313) had no effect on the CNO-mediated increase in paw withdrawal threshold (0.44 g, 95% CI [0.16, 0.72 g]).

**Figure 6.**
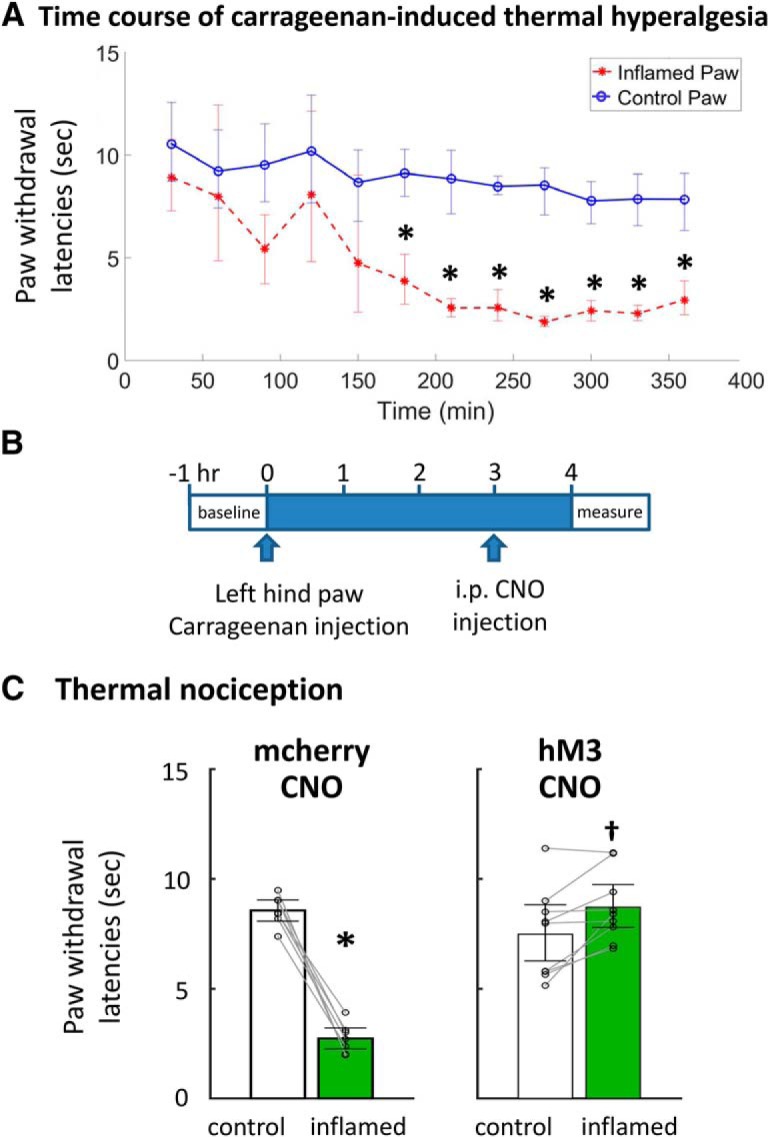
Effect of vlPAG dopaminergic neuron activation on carrageenan-induced thermal sensitivity. ***A***, A stable and significant reduction in paw withdrawal time to a thermal stimulus was achieved 180 min following carrageenan injection into the left hind paw of DAT-cre mice. ***B***, Pre-carrageenan paw withdrawal latencies were measured to obtain a baseline. Three hours after carrageenan injection, experimental and control mice received intraperitoneal CNO injections. One hour later, paw withdrawal latencies were recorded for the inflamed and control paws. ***C***, In mice expressing the hM3Dq DREADDs (hM3), CNO activation of vlPAG DA neurons produced an analgesic effect by significantly increasing the paw withdrawal latency of the carrageenan-inflamed paw (†, paired *t* test, df8, *p* = 0.0115). *p < 0.05.

Finally, carrageenan was injected into the hind paw of DAT-cre mice as a model for inflammatory pain to determine whether the antinociceptive effect of vlPAG DA activation was potent enough to inhibit persistent pain. Figure 6*A* shows the characteristic decrease in paw withdrawal thermal threshold in the carrageenan-treated hind paw, which reached steady state ∼3 h after injection. Baseline measurements were made before carrageenan hind paw injection. Three hours later, mice received intraperitoneal CNO injection followed, an hour later, by paw withdrawal latency measurements (Fig. 6*B*). Control mice who expressed mcherry but lacked DREADDs demonstrated a significant decrease in withdrawal latencies in the carrageenan-treated paw compared with the uninflamed paw (–6.13 s, 95% CI [–6.53, –5.45 s], paired *t* test, df6, *p* < 0.0001; Fig. 6*C*), and CNO administration did not produce any effect (–5.49 s, 95% CI [–7.02, –5.01 s], paired *t* test, df6, *p* < 0.0001). However, CNO activation of vlPAG DA neurons in mice expressing hM3 DREADDs induced an analgesic effect, significantly increasing the paw withdrawal latency of the carrageenan-inflamed paw (1.3 s, 95% CI [0, 2.16 s], paired *t* test, df8, *p* = 0.0115), indicating that activation of vlPAG DA neurons was sufficient to inhibit persistent inflammatory pain.

## Discussion

Here, we report that chemogenetic activation of vlPAG glutamatergic or dopaminergic neurons significantly attenuates both thermal and mechanical nociception, and that dopaminergic antinociception is prevented by the nonspecific DA receptor antagonist haloperidol. In addition, inhibiting either glutamatergic or dopaminergic neurons causes thermal and mechanical hypersensitivity. Despite similar antinociceptive effects, activating vlPAG glutamatergic neurons induced fear behaviors such as increased freezing time in the open field test and decreased light area exploration in the light/dark test, while there was no increase in fear behaviors with chemo-activation of vlPAG dopaminergic neurons. Finally, vlPAG DA neuron activation was sufficient to inhibit the persistent nociception caused by carrageenan-induced inflammation. These results demonstrate that there is a way to separate the generation of analgesia from anxiety when targeting the vlPAG, by selectively targeting DA neurons.

The antinociceptive and anxiogenic effects of vlPAG glutamatergic neurons have been studied for many years (Bandler et al., 1985; Bandler and Carrive, 1988; Jacquet, 1988; Jones and Gebhart, 1988; Jensen and Yaksh, 1989; Carstens et al., 1990; Tovote et al., 2016; Samineni et al., 2017). However, it was only recently that an amygdala-PAG-medullary circuit responsible for generating freezing behavior was elucidated (Tovote et al., 2016). A subpopulation of vlPAG glutamatergic neurons project to pre-motor cells located in the magnocellular nucleus (Mc) of the medulla and cause freezing when excited. Disinhibition of this vlPAG**→**Mc pathway occurs by way of a disynaptic GABAergic micro-circuit receiving inhibitory input from CEA. The CEA sends GABAergic projections to the vlPAG that preferentially target vlPAG GABAergic cells. These vlPAG GABAergic interneurons integrate multiple inhibitory and excitatory inputs and help to regulate the selection of either freezing or flight behaviors. Specifically, inhibition of the vlPAG GABAergic cells by GABAergic CEA inputs causes excitation of the vlPAG glutamatergic neurons that project to the Mc and produce passive, freezing behavior. In contrast, excitation by glutamatergic inputs from the dlPAG inhibits the projections to the Mc causing the animals to exhibit active, flight behavior.

Global activation of vlPAG glutamate neurons, as was achieved in the present study, also produces antinociception. However, as demonstrated by Tovote et al. (2016), there was no antinociceptive effect when excitation was restricted to the subpopulation of glutamate neurons projecting to the Mc, suggesting that another glutamatergic circuit is mediating the antinociceptive effects. Based on pharmacologic studies, a glutamatergic pathway appears to connect the vlPAG with the RVM and is directed at RVM off-cells specifically. Activation of these cells by morphine administration or any other means produces antinociception (Tortorici and Morgan, 2002; Morgan et al., 2008). These results strongly suggest that subpopulations of glutamatergic neurons within the vlPAG exert different effects based on the anatomic location of their projections.

We chose to use the known antinociceptive and anxiogenic effects of vlPAG glutamatergic activation as a positive control to compare and contrast the new observation that vlPAG/dorsal raphe dopaminergic neurons produce antinociceptive without anxiogenic behavioral effects. In contrast to vlPAG glutamatergic neurons, vlPAG dopaminergic neurons do not project to the RVM. Instead, they project up to the ventral tegmental area (VTA), the nucleus accumbens (NAc), the BNST, and CEA (Li et al., 2016). Recent studies demonstrate that direct VTA DA neuron stimulation attenuates neuropathic allodynia and activates exercise induced hypoalgesia (Kami et al., 2018; Watanabe et al., 2018). Therefore, vlPAG/dorsal raphe DA neurons may be exerting their antinociceptive effects by modulating DA levels in the VTA and NAc, rather than working through the classic descending inhibition pathway. Our studies also suggest the vlPAG/dorsal raphe neurons do not directly interact with the amygdala–PAG–medullary circuit, as these animals failed to show anxiogenic behavioral effects. Studies have been conducted to elucidate the electophysiologic effect of GABA neurons on vlPAG/DA function. However, no study has yet investigated the local microcircuit effects that these DA neurons exert within the PAG.

It is well known that VTA DA neurons are an essential part of the circuit mediating locomotion, and it has been demonstrated that chemogenetic activation of VTA DA neurons increases distances traveled in the open field test (Wang et al., 2013; Boekhoudt et al., 2016). However, vlPAG/dorsal raphe DA neurons do not appear to affect locomotion, as chemogenetic activation of vlPAG DA neurons failed to increase the distance traveled in the open field test, both in the present study as well as in studies by others (Li et al., 2016), suggesting that the decrease in freezing time and activity in the light/dark tests can be attributed to effects on anxiety behaviors rather than on movement directly.

Previous attempts to characterize the analgesic efficacy of vlPAG DA neurons stimulation have used acute, episodic pain stimuli including measuring hot plate latency and the tail flick test (Flores et al., 2004; Meyer et al., 2009; Li et al., 2016). In addition to acute, episodic pain, we have demonstrated that the analgesic effect is sufficient to decrease persistent inflammatory pain as well. However, additional studies are needed to determine whether the antinociception produced by activation of vlPAG DA neurons is sufficient to treat other forms of chronic pain, including neuropathic and cancer pain.

In this regard, while the use of chemogenetic or optogenetic techniques are not currently available as clinical treatments, the observation that selective DA neurons activation provides analgesia could be clinically significant. DA agonists and stimulants are commonly used in the treatment of Parkinson’s disease and attention deficit disorder, but are not currently used for the relief of pain. Pre-clinical studies in rodents suggest that DA agonists and psychostimulants such as d-amphetamine and methylphenidate (MPH), are effective analgesics and provide synergistic effects with opioids. Several groups have demonstrated that (1) DA agonists produce analgesia alone (Dennis and Melzack, 1983; Lin et al., 1989; Morgan and Franklin, 1990); (2) when combined with opioids, DA agonists potentiate opioid analgesia (Burrill et al., 1944; Goetzl et al., 1944; Ivy et al., 1944); (3) opioid analgesia is in part mediated through the actions of DA (Morgan and Franklin, 1991); (4) both D_1_ and D_2_ receptors are involved in the effect (Morgan and Franklin, 1991; Flores et al., 2004; Meyer et al., 2009); (5) the VTA contributes to both the rewarding and analgesic actions of DA (Morgan and Franklin, 1990; Matsui et al., 2014; Schifirneţ et al., 2014; Fields and Margolis, 2015; Trang et al., 2015); and (6) DA neurons in the ventral PAG play key roles in DA-mediated analgesia (Flores et al., 2004; Meyer et al., 2009; Chiou et al., 2013).

While we specifically targeted DA transporter-containing neurons, midbrain DA neurons have been shown to co-release neurotransmitters such as GABA or glutamate (Hnasko et al., 2010; Stuber et al., 2010; Tecuapetla et al., 2010; Tritsch et al., 2012; Li et al., 2016). It is therefore possible that the behavioral effects may not be purely DA mediated. To gain insight into this question, antagonists selective for DA receptor subtypes were given systemically to block the analgesic response. Previous studies showed conflicting results with regards to which DA receptor mediated PAG analgesic effects; one study suggested it was through D1 receptors (Flores et al., 2004) while another showed that D2 receptors were crucial (Meyer et al., 2009). In the current experiments, the antinociception produced by vlPAG DA neuron stimulation was incompletely prevented by selective blockade of DA D1 receptor or D2 receptor, and completely prevented by the non-specific DA receptor antagonist haloperidol, suggesting that both receptors may contribute to the antinociceptive effect.

Here, we show that selective stimulation of DA neurons within the vlPAG/dorsal raphe produced antinociception without anxiety, and further characterized a novel analgesic target, as there are no medications used clinically which specifically target dopaminergic circuits for the relief of pain.

## Synthesis

Reviewing Editor: Lorna Role, Stony Brook Decisions are customarily a result of the Reviewing Editor and the peer reviewers coming together and discussing their recommendations until a consensus is reached. When revisions are invited, a fact-based synthesis statement explaining their decision and outlining what is needed to prepare a revision will be listed below. The following reviewer(s) agreed to reveal their identity: Thomas Kash.

The authors' response to the reviewer's comments has partially addressed significant gaps in the manuscript. The changes that were introduced include a more thorough description of the methodology, the elimination of the inconsistencies in the statistical analysis, some additional data on the experimental validation of their chemogenetic approach, and further analysis of their data sets. These changes to strengthen the report.

Nevertheless, some important points were not addressed that must be dealt with. At eNeuro, we try to be very explicit about what needs to be done. As such, we request that all of the following issues are addressed in this final attempt at revision of the manuscript.

1. Quantification required:

The authors added two traces of electrophysiological recordings in response to the previous critiques which requested electrophysiological validation that the DREADDs were having the assumed effect on these neurons. Quantification of the effects on membrane potential and action potential firing need to be provided.

2. The authors should provide some additional discussion to put these findings in context with the literature.

In particular, it is important to address the divergence between the role of Vglut2 and DAT neurons. Anatomical description of these populations exist in the literature, for example, and could provide a framework to speculate as to why stimulation of these two population have similar anti-nociceptive properties but differ when referring to anxiety states.

3. The inconsistent use of controls in the manuscript makes it hard to compare not only between panels within the same figure/experiment but also between experiments.

The authors inconsistently utilize CNO and saline as interchangeable approaches to demonstrate that the DREADD effect is specific to administration of CNO in DREADD expressing animals. Although Figures #1 and #2 demonstrate that saline has no effect in mCherry animals, similar controls are not present for mechanical von Frey as done for the Hargreaves test.

a. In Figure #3, control animals are labeled as non-DREADD animals. If these are just naive animals, this would not be the appropriate control. This experiment needs mCherry expressing Vglut2 and DAT animals.

b. In Figure #4, panels A and B differ in the control being referred to as Baseline and Saline, respectively.

c. In Figure #5, panel C shows both Saline and CNO injections for the mCherry animals, but only CNO for the hM3Dq. What is the rationale for these differences in control groups?

4. The abstract still includes assertions that are beyond the scope of the data obtained.

a. The abstract indicates that D1/D2 receptors are shown to be critical based on the antagonist study. b. The last sentence still calls these ‘PAG DA neurons’, although the paper is mostly revised to indicate dorsal raphe may also be involved, given the targeting issues.

5. The reviewers found the authors response to other queries vis a vis controls to be less responsive Specifically we do not agree that because somebody else showed a particular virus worked in a particular mouse line in a particular region, that that is sufficient for all subsequent studies. Each of us is beholden to rigorously validate our viral tools and manipulations. In the least, we would expect some images to be included that show penetrance and fidelity of virus.
